# Cardio-Metabolic Disease and Polycystic Ovarian Syndrome (PCOS): A Narrative Review

**DOI:** 10.7759/cureus.25076

**Published:** 2022-05-17

**Authors:** Sai Lahari Sangaraju, Daniela Yepez, Xavier A Grandes, Ramya Talanki Manjunatha, Salma Habib

**Affiliations:** 1 Research, P.E.S. Institute of Medical Sciences and Research, Kuppam, IND; 2 General Medicine, Universidad Catolica de Santiago de Guayaquil, Guayaquil, ECU; 3 General Physician, Universidad Catolica de Santiago de Guayaquil, Guayaquil, ECU; 4 Graduate Medical Education, Kempegowda Institute of Medical Sciences, Bangalore, IND; 5 Medicine and Surgery, Institute of Applied Health Sciences (IAHS), Chittagong, BGD

**Keywords:** metabolic syndrome and endocrinology, cvd risk factors, pcos and cardio-metabolic consequences, cardio-metabolic diseases, polycystic ovary syndrome (pcos)

## Abstract

Polycystic ovarian syndrome (PCOS) is considered the most common endocrine disorder affecting females in today's world. Although it has been primarily studied and discussed in terms of its reproductive symptoms such as infertility, amenorrhea or oligomenorrhea, acne, hirsutism, and mood disorders, there is yet another unexplored and under-diagnosed category in the PCOS spectrum of diseases: its cardio-metabolic consequences. PCOS patients are prone to these abnormalities from a very young age, increasing their morbidity and mortality rates compared to their regular counterparts. The usual pathogenesis of PCOS is a culmination of several genetic and environmental factors. Regarding its cardio-metabolic aspects, insulin resistance (IR) is said to be the single most important cause of a variety of metabolic risk factors, including type 2 diabetes mellitus (T2DM), metabolic syndrome (MetS), dyslipidemia, obesity, and hypertension (HTN), whereas a few other non-traditional factors such as C-reactive protein (CRP), carotid intima-media thickness (IMT), coronary artery calcification (CAC), and endothelial dysfunction are also said to be increased in PCOS patients, further increasing their risk of complications due to cardiovascular diseases (CVD). A timely diagnosis and adequate treatment of these risk factors by using lifestyle interventions, diet, and/or medications are essential to reduce the burden of PCOS in today's world. This article has highlighted an array of traditional and non-traditional cardio-metabolic consequences PCOS patients are prone to and their systematic pathogenesis. In addition, an outline of recommendations has been given in the pharmacological and non-pharmacological sections of this article, which may benefit doctors in managing this challenging condition.

## Introduction and background

**Figure 1:**
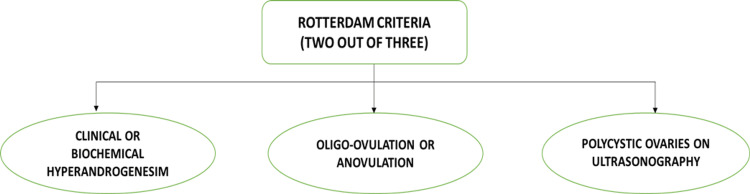
Figure depicting the diagnostic criteria for PCOS. PCOS: polycystic ovarian syndrome. Image credits-Sai Lahari Sangaraju.

Stein and Leventhal first described polycystic ovarian syndrome (PCOS) in the year 1935 in the form of a report named "Amenorrhoea associated with polycystic ovaries," which from then on, has led to a plethora of research in the field of medicine [1]. PCOS is a common but complicated endocrine disorder that affects 6%-10% of females in the reproductive age group all over the world [2]. PCOS is most often defined and diagnosed according to the 2003 Rotterdam criteria, which mandate the presence of two of the following three clinical features (Figure 1) [3].

**Figure 2:**
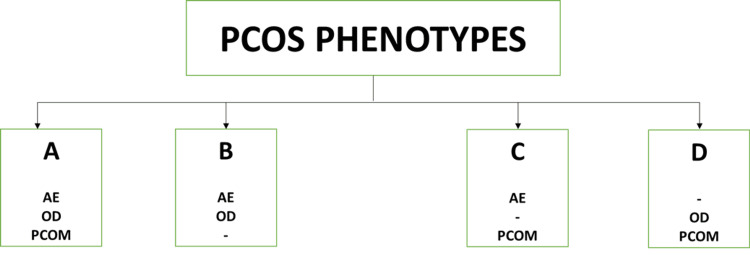
Figure depicting the four phenotypes of PCOS. PCOS: polycystic ovarian syndrome, AE: androgen excess, OD: ovulatory dysfunction, PCOM: polycystic ovarian morphology. Image credits-Sai Lahari Sangaraju.

Innumerable attempts were made to classify PCOS. Finally, in 2012, the National Institute of Health (NIH) consensus panel decided on four PCOS phenotypes (A, B, C, and D). Phenotypes A and B were termed as "classic PCOS," patients with features of hyperandrogenism, ovulatory dysfunction, and polycystic ovarian morphology were included in phenotype A, whereas patients with only hyperandrogenism and ovulatory dysfunction were categorized as phenotype B. Phenotype C was termed "ovulatory PCOS" and included patients with hyperandrogenism and polycystic ovaries. Lastly, phenotype D was termed non-hyperandrogenic PCOS, and it included patients with polycystic and dysfunctional ovaries (Figure 2) [4].

Among the four phenotypes of PCOS, classic PCOS patients demonstrated higher insulin levels, greater rates of IR, higher body mass index (BMI), and a higher incidence of obesity, whereas patients with "ovulatory PCOS" had milder clinical and endocrine changes. Similarly, when compared to patients with classic PCOS, "non-hyperandrogenic" PCOS patients showed lower luteinizing hormone (LH) to follicle-stimulating hormone (FSH) ratios and greater sex hormone-binding globulin levels [5-7]. 

However, the exact cause of PCOS is yet to be found. A study was done in the Hainan province in four cities, namely, Lingshui, Qiongzhong, Changjiang, and Sanya. It narrowed down the risk factors to irregular menstrual cycles, a family history of diabetes and infertility, a history of irregular menstruation in the mother, lack of regular exercise, and a distressed mood [8]. Furthermore, all of these risk factors primarily affect the physiology of the ovaries, the hypothalamic-pituitary axis, and insulin metabolism [9,10]. The current literature also suggests an autosomal dominant pattern of inheritance in PCOS female patients [9]. Approximately 20-40% of first-degree female relatives of PCOS patients are diagnosed with the disorder, compared to a 4-6% probability in the general population [11]. PCOS is a disorder that can affect females right from their fetal life up until their death, with several complications in between, that affect their mortality and morbidity as well as decrease their quality of life [12]. If affected by PCOS, the therapy goals are tailored according to each patient's needs. In general, lifestyle modifications are of utmost importance, including dietary changes, regular exercise, and optimum weight maintenance. These can be included with pharmacological drugs such as metformin, lipid-lowering agents, and oral contraceptive pills (OCPs) [13]. Premature recognition of high-risk populations through rampant screening and early treatment of dreadful complications like cardio-metabolic abnormalities is pivotal to decreasing the burden of the disease [14]. Despite the lack of data connecting cardio-metabolic events and mortality in PCOS patients, the related risks have been identified well [15]. They include insulin resistance (IR), glucose intolerance, dyslipidemia, sub-clinical atherosclerosis, and vascular abnormalities with no regard to BMI [16]. However, if obese, the metabolic risk factors may be amplified, which can, in time, lead to an increased probability of worse cardiovascular outcomes [15]. This review article explores the spectrum of cardio-metabolic implications PCOS patients are predisposed to in the long run and highlights the treatment options that can help them have a better chance at life.

## Review

**Figure 3:**
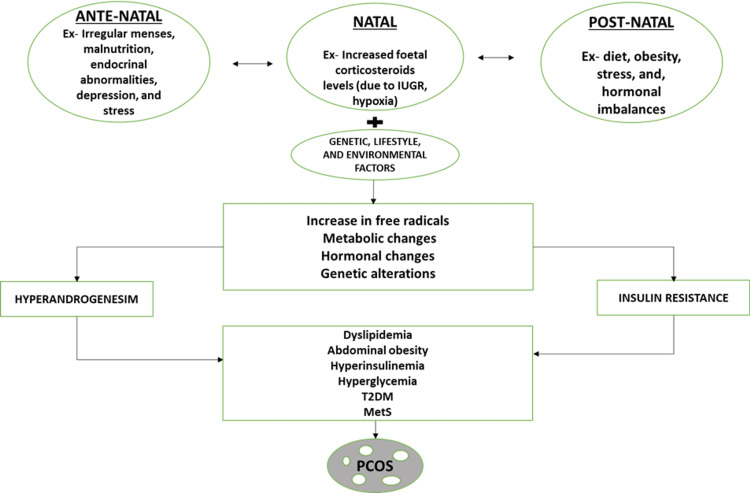
Summary of the pathogenesis of PCOS. Ex: example, IUGR: intrauterine growth restriction, T2DM: type 2 diabetes mellitus, MetS: metabolic syndrome, PCOS: polycystic ovarian syndrome. Image credits-Sai Lahari Sangaraju.

With recent research, it has become undeniable that PCOS is a complex culmination of symptoms of various organ systems of the body with innumerable complications, including grave cardio-metabolic risks, affecting female patients throughout their life cycle [14]. The development of the syndrome appears to be caused by a combination of genetic predisposition and prenatal and postnatal environmental factors such as obesity, diet, stress, and endocrine abnormalities. Excess corticosteroids in the new-born, either due to excess glucocorticoids in fetal life or due to intrauterine growth restriction (IUGR) and excess androgens in the mother due to endocrine disturbances, can predispose to conditions such as oxidative stress, low-grade chronic inflammation, and changes in insulin and lipid metabolism, all of which are present in the background of the syndrome's main clinical manifestations: hyperandrogenism, anovulation, and polycystic ovary morphology, which also serve as the diagnostic criteria in PCOS patients (Figure 3) [12].

Cardio-metabolic risk factors and associated sub-clinical-clinical outcomes of PCOS

Insulin Resistance and Type 2 Diabetes Mellitus

IR, by definition, is the inefficiency of insulin to guide glucose systematically into the body cells [17]. IR usually occurs due to the defect in post-binding insulin signaling due to an increase in serine phosphorylation, in addition, defective tyrosine phosphorylation of insulin receptors and insulin receptor substrate-1 can also cause metabolic disturbances in skeletal muscle, adipocytes, and the ovaries. Reduced glucose transporter 4 (GLUT4) in subcutaneous adipocytes, impaired insulin clearance in the liver, mitochondrial functional abnormalities, and serine kinase stimulation in the mitogen-activated protein kinase/extracellular signal-regulated kinase (MAPK-ERK) pathway are some alternate mechanisms of IR that can lead to an increased risk of T2DM [18]. In a review study done by Ovalle et al., it was stated that 50-80% of PCOS patients had a higher probability of developing IR, and in a prospective controlled study in 254 PCOS patients, impaired glucose tolerance (IGT) and DM2 were found to be more common in patients with PCOS, with prevalence rates of 31.3% and 7.5%, respectively, compared to 14% for IGT and 0% for DM2 in age-and weight-matched non-PCOS controls [19,20]. IGT has recently also been linked to an increased risk of cardiovascular disease (CVD), mortality, and T2DM among the general public [21]. A population-based study from 2005 cited that the mortality rate of people with IGT was 5.5% over five years, whereas it was 1.9% in people with no IGT [21]. A large cohort-based study conducted in the general Australian population displayed the conversion rate of IGT to T2DM to be 2.9% per year in young females, whereas another study in the same nation demonstrated a higher rate of conversion at 8.7% per year over 6.2 years in PCOS patients [21,22]. After significant research on PCOS and T2DM, the International Diabetes Federation named PCOS a non-modifiable risk factor for diabetes mellitus [23].

Thus, PCOS patients have to further face all the associated risks of IR like acanthosis nigricans, premature adrenarche, hyperandrogenemia, hirsutism, irregular menstrual cycles, central obesity, dyslipidemia, hypertension (HTN), microalbuminuria, hypercoagulable state endothelial dysfunction, glucose intolerance, T2DM, and early cardiovascular and cerebrovascular disease when compared to their regular counterparts, making the diagnosis and timely management of IR in PCOS patients a dire need [19].

Metabolic Syndrome 

MetS, by definition, include hyperglycemia (fasting glucose levels 5.6 mmol/L or above), low high-density lipoprotein (HDL) (<1.29 mmol/L), high total triglyceride (TG) level (1.7 mmol/L or above), central obesity (increased waist circumference tailored to the given population and country of residence), and increased systolic or diastolic blood pressure (130/80 mmHg or above) [24]. The development of MetS in PCOS patients can be accounted to IR. All clinical outcomes included in MetS have various attributable mechanisms; for example, HTN arises from endothelial damage and reduced nitric oxide bioavailability, and dyslipidemia develops from IR via increased secretion of non-esterified fatty acids and raised TG production. Furthermore, IR can also cause hyperglycemia to evolve due to compensatory hyperinsulinemia and pancreatic beta-cell depletion [25,26]. MetS ultimately is a culmination of various metabolic components that already belong to the established risk factors sector of cardio-vascular diseases like IGT, IR, T2DM, dyslipidemia, obesity, and HTN [27]. The odds of MetS occurring in PCOS patients is higher when compared to the normal population. In 2010, a meta-analysis involving 2256 PCOS patients and 4130 controls was undertaken. MetS displayed a prevalence odds ratio (OR) of 2.88 (95% CI 2.40-3.45) with significant heterogeneity (I2 = 67%, p < 0.0001). Further, subgroup research of five studies was undertaken in the same study, involving 273 PCOS patients and 276 controls; here, the OR was 2.20 (95% CI 1.36-3.56) with no evidence of significant heterogeneity (I2 = 38%, p = 0.17) [27]. Surprisingly, even teenagers with PCOS frequently have IGT, T2DM, and MetS, implying that PCOS harms metabolic health throughout a female's lifespan and thus increases the burden of the already existent futile diseases like T2DM, HTN, and CVD in society [28].

Dyslipidemia

**Figure 4:**
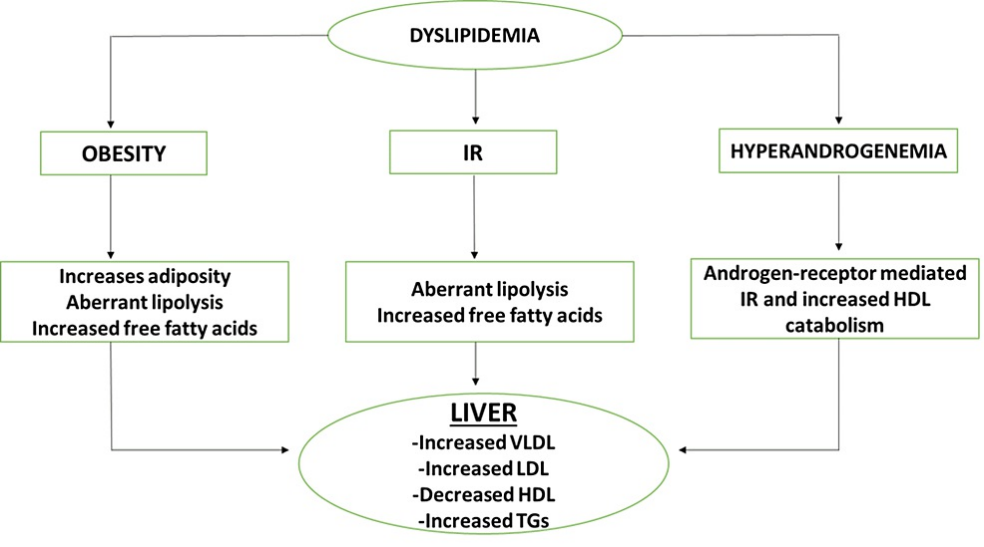
Summary of the mechanism behind dyslipidemia. IR: Insulin resistance, VLDL: very-low-density lipoprotein, HDL: high-density lipoprotein, LDL: low-density lipoprotein, TGs: triglycerides. Image credits-Sai Lahari Sangaraju.

Dyslipidemia by definition includes, low-density lipoprotein (LDL) cholesterol concentration >160 mg/dL, HDL cholesterol concentration <40 mg/dL, TG >200 mg/dL, or use of medications that aid in reducing cholesterol levels [29]. These lipid abnormalities are linked to IR and have been shown to predict the onset of CVD such as myocardial infarction (MI) [30]. The interconnected consequences of obesity, IR, and hyperandrogenism are thought to cause dyslipidemia in PCOS patients. IR, overproduction of very-low-density lipoprotein (VLDL), aberrant lipoprotein lipase-mediated lipolysis, and a malfunction in the insulin-signaling pathway mediated by overexpression of the PI3KR1 gene are among the mechanisms through which increased adiposity occurs, which in turn links dyslipidemia to PCOS. Excessive hepatic synthesis of apoB-containing VLDL is associated with IR and hypertriglyceridemia. Excess testosterone levels in PCOS patients can also be the reason behind dyslipidemia as increased levels of testosterone cause androgen receptor-mediated IR and overexpression of genes involved in HDL catabolism, which is considered to be a good form of cholesterol (Figure 4) [31]. 

A prospective study was done by Wang et al. among 1,127 females, of which 53 (4.7%) aged 20-32 met the criteria for PCOS, observed a dramatic two-fold increase in the incidence of dyslipidemia in PCOS patients when compared to healthy females over a time frame of 18 years [32]. Similarly, in a meta-analysis of 30 studies that matched females by weight, female patients with PCOS had 12.6 mg/dL higher LDL cholesterol, 18.8 mg/dL higher non-HDL concentrations, 26.4 mg/dL higher TG, and 6.4 mg/dL lower HDL cholesterol levels compared to age-matched females who did not have PCOS [30]. When matched by BMI, the same females had 9.2 mg/dL and 16.3 mg/dL higher LDL cholesterol and non-HDL cholesterol values than healthy females [30]. Hence, the frequency of dyslipidemia can be higher in PCOS patients. PCOS patients had greater LDL-cholesterol and non-HDL-cholesterol, regardless of BMI, and known TG and HDL cholesterol changes. All PCOS patients should be evaluated for dyslipidemia, including LDL and non-HDL cholesterol levels, for optimal cardiovascular risk prevention.

*Obesity* 

Obesity is a concurring risk factor in PCOS patients. Studies show that IR is the root cause of obesity because it causes hyperinsulinemia, which stimulates steroid synthesis from the ovary and adipose tissue, which in turn reduces the hepatic secretion of sex hormone-binding globulin, causing free androgens to rise, which, if increased chronically, can lead to central obesity via accumulation of visceral fat, worsening symptoms of PCOS and thus, creating a vicious cycle of complications [33,34]. Alvarez-Blasco et al. demonstrated the prevalence of PCOS in overweight (BMI: 25.0-29.9 kg/m²) and obese (BMI ≥30 kg/m²) female patients to be at 28.3%, whereas there was a 5.5% prevalence in lean females [35]. A meta-analysis was conducted on 15,000 females; it was observed that PCOS patients had an increased probability of overweight Risk Ratio (RR) 1.95 (95% CI 1.52-2.50), obesity (RR 2.77 (1.88-4.10), and central obesity (RR 1.73 (1.31-2.30)) in comparison with healthy females [33]. Conversely, in other regions of the globe, the burden of obesity in PCOS patients is 30% less when compared to the USA, raising a question of cultural and ethical variance [36]. This relationship was further strengthened by another study that stated that Caucasian females with PCOS had an increasingly significant relationship with obesity (RR 10.79 (5.36-21.70)) when compared to Asian females (RR 2.31 (1.33-4.00)) [33]. Furthermore, a study done in China on PCOS patients reported normal BMIs, supporting the existing disparity among different populations [37]. Despite having a low BMI, they can still have cardio-metabolic complications [38]. Based on IR being the key pathogenic reason behind obesity, central obesity in a PCOS patient also worsens all the insulin-related metabolic complications such as hyperandrogenemia, menstrual irregularities, dyslipidemia, HTN, and T2DM, making them more prone to cardiovascular and cerebrovascular diseases [19]. Thus, obese PCOS patients are more likely to have it worse with regards to their reproductive and overall clinical picture in comparison with their leaner counterparts; hence, they are more likely to be diagnosed and managed earlier, which can supposably be the reason behind the exaggerated link between obesity and PCOS [33]. 

Hypertension

American College of Cardiology (ACC)/American Heart Association (AHA) defined HTN as systolic blood pressure (SBP) greater than or equal to 130 mmHg and diastolic greater than or equal to 80 mmHg; using these set parameters, HTN had a 24% higher prevalence in PCOS patients when compared to healthy females [39]. The stimulation of the renin-angiotensin system causes HTN in PCOS patients [40]. Previous research has found that PCOS patients have greater aldosterone levels than age-and BMI-matched controls [40]. Furthermore, an imbalance in the autonomic nervous system increased renal sodium reabsorption, and a reduction in the synthesis of nitric oxide have all been linked to the onset of HTN in female patients with PCOS [39]. In a cross-sectional study, among 233 female patients with PCOS and 70 controls, the chances of HTN in patients with PCOS were 65% compared to 41% in non-PCOS females [39]. However, a 12-year follow-up study on 637 participants showed similar HTN incidence in PCOS and non-PCOS females [41]. As far as comparisons within PCOS groups, a longitudinal survey by Huddleston et al. depicted an increase in blood pressure every year or from a baseline value, and out of the two, systolic and diastolic, systolic blood pressure has an increased tendency to cause the rise in blood pressure [42]. PCOS patients with HTN can have an altered endothelial function (as measured by flow-mediated dilation of the brachial arteries), IMT at the carotid artery, and abnormal serum endothelin-1 values, suggesting early functional, structural, and biochemical pre-atherosclerotic vascular impairment [43]. Thus, HTN in PCOS patients can predispose them to vicious CVD outcomes such as atherosclerosis and MI, which will increase morbidity and mortality rates among them and increase the prevalence of such diseases in any given community. 

**Figure 5:**
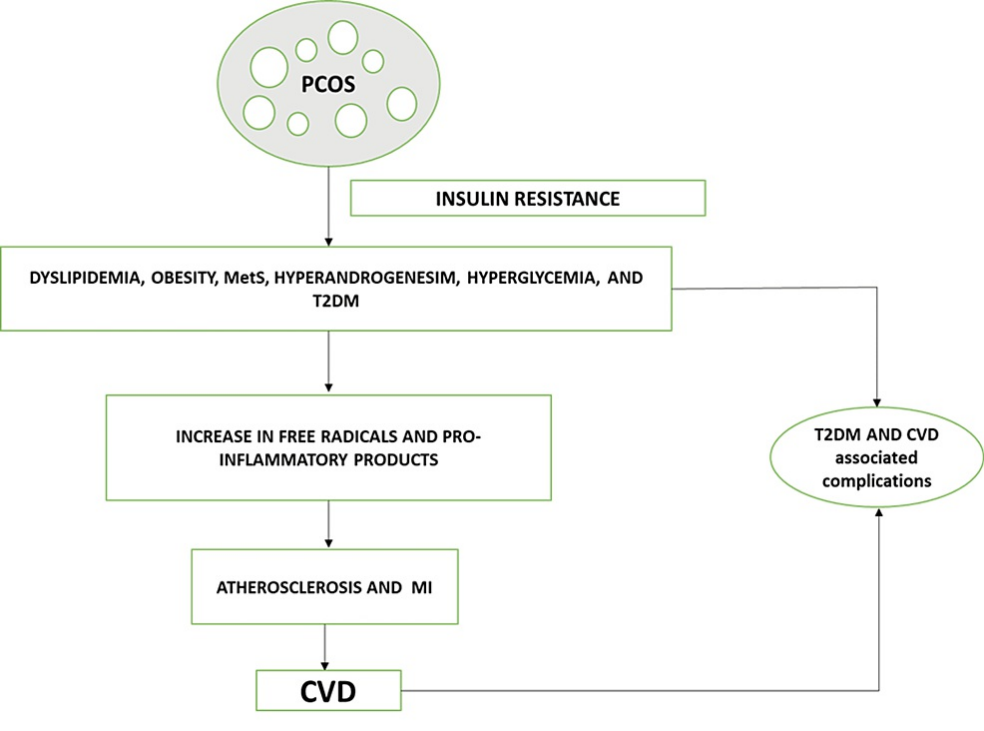
Summary of cardio-metabolic risk factors of PCOS. PCOS: polycystic ovarian syndrome, MetS: metabolic syndrome, T2DM: type 2 diabetes mellitus, MI: myocardial Infarction, CVD: cardiovascular disease. Image credits-Sai Lahari Sangaraju.

Thus, drawing conclusions from the discussed risk factors, PCOS patients have been found to have a wide range of probable cardio-metabolic disease outcomes, which have been summarized below (Figure 5). 

Sub-clinical-clinical CVD outcomes 

**Figure 6:**
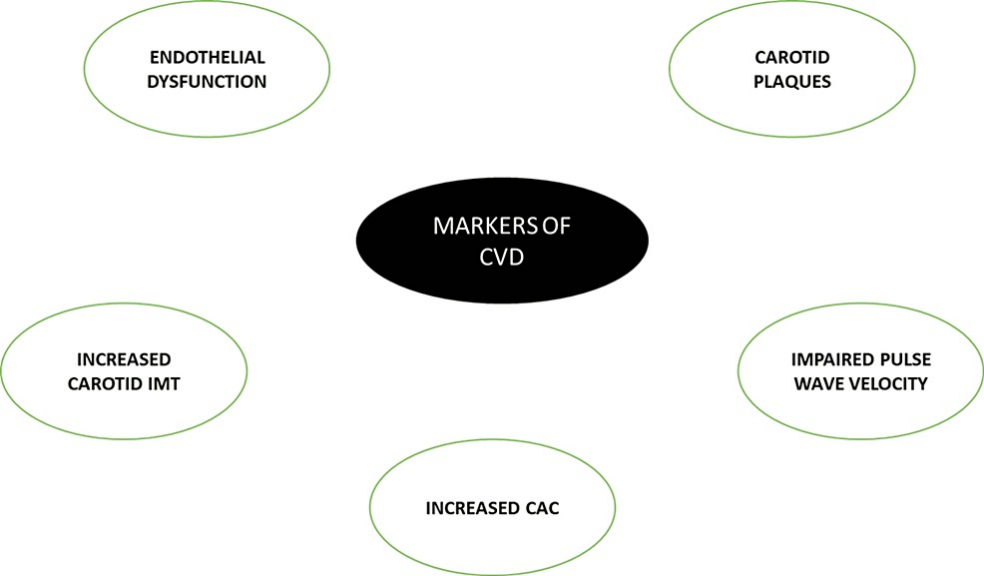
Summary of various markers of CVD. CVD: cardiovascular disease, IMT: intima-media thickness, CAC: coronary artery calcification. Image credits-Sai Lahari Sangaraju.

PCOS patients are said to be at an increased risk for CVD, given that up to 65% of CVD deaths occur in people with IGT, and since IGT and T2DM risks are higher in female patients with PCOS, it is reasonable to assume that PCOS patients have a higher CVD risk [21]. The cardio-vascular aspect of PCOS can be explored and identified with the help of sub-clinical and clinical markers such as endothelial dysfunction, impaired pulse wave velocity, increased carotid IMT, carotid plaques, and increased CAC (Figure 6) [44,45]. 

Sub-clinical non-invasive markers of atherosclerosis, such as CAC and carotid IMT, have consistently been linked to the future prevalence of CVD in prospective epidemiologic studies. According to one study, the frequency of CAC in older females with PCOS aged 40-61 years was 46% vs 31% in controls, and the mean (SD) CAC score was substantially higher in PCOS patients than in controls (21.7 (108.6) vs 3.7 (16.0), p = 0.033) [46]. Additionally, PCOS was still a predictor of CAC after adjusting for age and BMI (OR: 2.31 (1.00-5.33)) [46]. In a prevalence study, among 143 female subjects who underwent coronary angiography, patients with extensive coronary artery disease were found to have polycystic ovaries on ultrasonography [47]. Another novel adipokine marker, omentin, can be beneficial in assessing CVD risk. It is produced chiefly by the visceral adipocytes and is a chief contributor to the atherosclerotic processes, thus establishing a connection between metabolic abnormalities like obesity and diabetes as in PCOS to CVD. In a study done among 60 patients of T2DM with or without carotid disease and controls, serum omentin levels and other cardiovascular risk factors were assessed, and omentin levels were found to be decreased in T2DM patients, predominantly in patients with carotid plaques. As a result, omentin could be a potential marker to predict CVD outcomes in PCOS patients, where an exceptional number of individuals are obese and diabetic [48].

Furthermore, there has been conflicting evidence as to whether PCOS raises the risk of clinical CVD events. A study used Taiwan National Health Insurance data to identify 8,048 females aged 15-49 years with PCOS and 32,192 females without the syndrome as controls. After a 5.9-year follow-up period, the incidence of coronary artery disease in PCOS females was found to be 63% greater. Additionally, in females with already present cardiovascular comorbidities, coronary artery disease's risk increased further [49]. A 2017 meta-analysis of eight studies involving 128,977 females aged 36-71 years with an average follow-up period of 10-40 years found that PCOS was linked to a higher risk of stroke (OR 1.36 (95% CI 1.09-1.70); p = 0.007) [50]. After controlling for BMI, however, this effect was weakened and no longer statistically significant (OR 1.24 (0.98-1.59) p = 0.077) [50]. In another meta-analysis found substantial relative risks of coronary heart disease of 1.26 for MI and 1.32 for stroke [51]. In addition, a meta-analysis of five case-control and five cohort studies involving over 100,000 females aged 20-74 years and 7-40 years of follow-up found no significant link between PCOS and MI (OR 1.01 (0.68-1.51); p = 0.95) [52]. Whereas, in terms of the physiology of the heart, PCOS patients have a lower cardiac systolic flow velocity than age-matched controls, and there is an inverse association between serum fasting insulin and left ventricular systolic outflow parameters [53]. Furthermore, PCOS patients are more likely to have a lower left ventricular ejection fraction and diastolic dysfunction [54,55]. A considerable increase in the left ventricular mass index, a predictor of CVD morbidity and mortality, has also been discovered in PCOS patients of normal weight [55]. Overall, studies comparing the cumulative CVD risk in PCOS patients vs the general population have also yielded varied results. For example, a follow-up study in Denmark spread over 11 years has concluded that PCOS patients have an increased propensity for CVD than their non-PCOS counterparts. The rates of CVD-associated events were 22.6 vs 13.2 per 1,000 patient-years among PCOS vs control females [56]. In contrast, female patients with PCOS had no increased risk of unfavorable cardiovascular events in midlife despite weighing more than controls in a study among 309 female patients with PCOS and 343 controls with a mean (SD) follow-up period of 23.7 (13.7) years [57]. The adjusted HR for MI and stroke, for example, was 0.74 (0.32-1.72); p = 0.48 and 1.05 (0.28-3.92); p = 0.94, respectively [57]. Thus, PCOS patients demonstrate an increased incidence of CVD risk factors than non-PCOS females. However, independent CVD risk in PCOS patients remains unclear and demands significant research. With the increased incidence of risk factors, the chance of screening, diagnosing, and providing early treatment is higher and can most likely reduce the overall burden. All patients should have their BMI, waist circumference, serum lipid/glucose, and blood pressure checked as part of their routine cardiovascular risk assessment. Paraoxonase, a high-density new lipoprotein-related enzyme-linked to increased oxidative stress, whose levels are claimed to be reduced in PCOS in patients with BMI ≥25kg/m², could serve as an emerging marker for PCOS, particularly in obese patients. Oral glucose tolerance (OGT) testing should be done in those who are obese, older, have a history of gestational diabetes, or have a risk of hereditary diabetes mellitus [58,59].

Management 

A heterogenic disease like PCOS can be considered a consequential epidemiological issue with a significant monetary burden. According to USA data, the burden of PCOS in Australia can be accounted for AU$ four billion [60,61]. Premature recognition of at-risk populations through rampant screening and early treatment of dreadful complications is pivotal to decreasing the burden of the disease [14]. According to the recently released international PCOS recommendations, PCOS patients should have their global CVD risk assessed regularly. Depending on the presence of diabetes risk factors, fasting blood glucose or glycosylated hemoglobin should be measured at the initial appointment and subsequent follow-up visits [62]. OGT testing should be used to assess people with CVD risk factors such as obesity, IGT history, gestational diabetes, diabetes in the family, or HTN. Lipid profiles, blood pressure, and weight should be checked regularly [38]. Drawing conclusions from the discussed clinical feature spectrum of PCOS, it is clear that no PCOS patient can have the same treatment plan; it should be highly personalized and symptom-specific to tackle the disease efficiently. According to their clinical presentation, female patients with PCOS can be treated via non-pharmacological and pharmacological approaches.

Non-pharmacological approaches

A nutritionally appropriate, low-fat diet (about 30% of calories, saturated fat around 10%), moderate protein (15%), and high carbohydrate consumption (nearly 55%), with increased fiber-rich whole-grain bread, cereals, fruits, and vegetables, and moderate regular exercise is recommended. Over 6 to 12 months, a modest energy restriction diet (500 to 1,000 kcal/day) decreases body weight by 7% to 10%. Fruit juice, soft drinks, and high-fat foods are to be avoided. These are a few simple and practical ideas that may be discussed in a few minutes in a medical consultation. In comparison to diet alone, incorporating simple, moderate physical activity, such as scheduled exercise (at least 30 minutes/day) and incidental exercise, improves weight reduction and clinical outcomes of PCOS [63]. In a two-year study, 307 obese people (mean BMI 36 kg/m²) were asked to weigh in on the carbohydrate versus low-fat diet controversy. The research participators were assigned at random to either a low-carbohydrate diet with no restrictions on fat consumption or a low-fat diet with about 30% of calories coming from fat. The methodology for both arms of the trial includes behavioral interventions such as self-monitoring and physical activity sessions. Participants from both groups lost a similar amount of weight. The majority of the lipid profile was similar in both groups. Although the low-carbohydrate group had significantly higher HDL cholesterol levels, the absolute difference was 2 mg/dL. The inclusion of men and the exclusion of those with diabetes and hyperlipidemia reduced the generalizability of PCOS. After two years, the 50% dropout rate reflects the difficulties of changing one's diet and lifestyle [64]. However, even as small as 5-10% weight loss has been proven to modify the outcomes of PCOS-associated abnormalities such as CVD, endocrine disturbances, and T2DM [65]. 

Pharmacological approaches

PCOS disease spectrum specifically includes chronic anovulation, hyperandrogenism, and clinical symptoms such as irregular menstrual periods, infertility, hirsutism, and acne. As a result, while one might focus on the metabolic and cardiac aspects of PCOS in clinical practice, pharmacological therapy is usually more of a symptom-specific approach and should never be used as an alternative to lifestyle modifications. Certain treatments aimed at alleviating these symptoms, on the other hand, may have negative consequences for other features of the illness, particularly the cardiometabolic ones. As a result, various therapy options for PCOS patients, with a focus on the cardiometabolic aspect, have been briefly discussed.

Low-Dose OCPs and Anti-androgens for Cardio-Metabolic Disturbances

For decades, OCP has been the cornerstone of PCOS pharmaceutical treatment. OCPs are more effective in changing menstrual patterns and lowering serum testosterone levels than any other medication, including insulin sensitizers and insulin-lowering drugs. Although some studies suggest adverse metabolic effects such as increased IR in people who use particular OCPs, meta-analysis does not support a link between OCP usage and poor metabolic profiles, and research outcomes may vary depending on the progestin used [66]. The progestogen component of OCP has been associated with dyslipidemia in OCP users [67]. Although some of the OCPs looked to elevate HDL levels significantly [67,68], the magnitude of the impact was generally small, and there was significant variation between the cohorts studied [69]. The usage of the OCP and the adverse effects it has on metabolic markers is vital in obese PCOS patients. The OCP (35 mg Ethinyl estradiol/2 mg cyproterone acetate), the most often administered OCP, enhanced IR by 25% in a recent randomized, controlled study [68]. More critically, greater IR was linked to an increase in arterial stiffness, which is a predictor of cardiovascular risk, emphasizing the significance of individualized medical care for PCOS patients, particularly those who are obese [68]. However, a low-dose of the oral contraceptive (OC) (20 g Ethinyl estradiol/100 g levonorgestrel with 50 mg spironolactone twice a day) did not affect IR and did not worsen arterial stiffness as determined by pulse wave velocity in the same trial. If contraception is required, a low estrogen formulation may be desirable, and a combination with an anti-androgen with established blood pressure effects, such as spironolactone, appears to be tolerated. Thus, more research is needed because studies are insufficient and the evidence is contradictory. However, the cardiometabolic effects of pharmacological therapy should be considered, and low-dose OCP formulations may be a superior alternative with equivalent efficacy and fewer cardiometabolic effects [68]. Spironolactone is an anti-androgenic medication used by PCOS patients. Since the 1950s, this aldosterone antagonist has been utilized as a potassium-sparing diuretic in the treatment of HTN. In investigations comparing the effects of spironolactone and metformin on blood pressure, no difference in blood pressure was observed with either therapy [70]. Whereas, in another study, mean blood pressure reduced dramatically from 118 ± 5/82 ± 4 mmHg to 113 ± 4/72 ± 5 mmHg (p<0.05) in patients treated with spironolactone 100 mg daily for two months [71]. Flutamide, another anti-androgen agent, has improved lipid profiles and adipokine levels [72]. According to Gambineri et al., PCOS patients on flutamide had lower visceral fat content, improved insulin sensitivity, and lower LDL cholesterol levels [73]. These findings are intriguing, especially because IR, obesity, and hyperandrogenemia are linked to PCOS cardiovascular risk. Thus, in treating overweight-obese PCOS females, there appears to be a reason for targeting distinct therapeutic approaches according to the desired long-term goals.

Anti-insulin Agents for IR and T2DM

According to several experts, IR, namely hyperinsulinemia, leads to hyperandrogenemia and is linked to cardiovascular and metabolic risk factors. Hyperinsulinemic-euglycemic clamp techniques use an intravenous insulin infusion to maintain constant serum glucose concentrations at fasting levels to help evaluate glucose uptake. Insulin resistance, if present, is indicated by lower glucose absorption. The procedure is experimentally useful but clinically inconvenient because it needs intravenous infusions and regular blood drawings and is time and money consuming. Other sophisticated testing methods that include intravenous insulin infusions (insulin sensitivity and insulin tolerance tests) have been attempted. However, they do not alleviate the time, financial, and testing burdens to make them relevant for widespread clinical practice, and normal cutoffs are not widely disseminated. Other approaches to IR evaluation have been validated using clamp techniques as comparisons [74].

Fasting methods to evaluate IR have been recommended as an addition to diabetes mellitus screening for many years. Fasting insulin levels of more than 20 U/mL may indicate IR. The fasting glucose/insulin ratio (G/I) has also acquired popularity in the medical community. In some populations, a ratio of less than 4.5 has been demonstrated to be more than 90% sensitive. The homeostatic model assessment (HOMA), a more complicated fasting computation, also yields good results. HOMA is calculated by dividing fasting glucose (mg/dL) and insulin (U/mL) by a constant [74]. At the same time, an OGT test, fasting plasma glucose (FPG), or hemoglobin A1c (HbA1c) can be used to determine the glycemic status of PCOS patients [75]. The OGT test is considered to be the gold standard test for T2DM diagnosis, and according to the American Diabetes Association, the cut-off value is ≥ 11.1 mmol/l [76]. Metformin is the most widely researched insulin-lowering medication in the treatment of PCOS. For 20 years, metformin, a biguanide antihyperglycemic, has been utilized to treat PCOS [69]. Metformin enhances insulin sensitivity by decreasing gluconeogenesis and lipogenesis and improving glucose metabolism in the liver, skeletal muscle, adipose tissue, and ovaries [77]. Metformin also lowers CRP levels, lowering CVD risk [78]. Metformin reduced blood pressure, fasting glucose, and serum androgens with no effect on body weight or hirsutism, according to a recent systematic meta-analysis based on small numbers compared it to placebo or no treatment [79]. In contrast, other studies have found that metformin reduces BMI in PCOS patients, which is consistent with evidence from the general population or those at risk of diabetes [80]. Although early studies suggested that metformin might be the best treatment for anovulation in PCOS. However, it is now thought that metformin may be the least beneficial in those who are highly obese (BMI greater than 35 kg/m^2^) [81]. Finally, a recent Cochrane Systematic Review comparing insulin-sensitizing drugs vs the combined OCP in PCOS patients found that metformin was more effective than the OCP in lowering fasting insulin and TG. However, there is limited data on the effects of lowering fasting glucose on cholesterol levels [82]. Furthermore, OCPs were linked to a better menstrual pattern and lower blood testosterone levels compared to metformin. The authors determined that evidence on important clinical outcomes such as developing diabetes, CVD, and endometrial cancer was inadequate or non-existent. According to the review, no trials compared metformin vs the OCP in terms of cardiovascular outcomes, such as stroke and MI, and only one trial compared metformin vs the OCP in terms of T2DM development, with no difference between the two groups; there was also no difference in BMI or waist/hip ratio between the metformin and OCP treatment groups [82]. However, only a few major randomized controlled trials in the literature show the efficacy of metformin in treating PCOS, and those that do exist reveal only moderate benefits of metformin treatment over alternative therapies [83]. 

Anti-obesity Agents and Bariatric Surgery for Obesity in PCOS

Orlistat, a pancreatic lipase inhibitor that inhibits dietary fat absorption, has recently been found to lower body weight and total testosterone levels in PCOS patients [84]. Additionally, orlistat treatment in PCOS patients improved IR indices and hormonal and metabolic profiles and reduced advanced glycation end-products after six months of treatment, regardless of BMI alterations [85]. As a result, orlistat could be a valuable adjuvant in PCOS treatment. Another study found that when metformin and orlistat were used in obese female patients with PCOS, they had similar effects on weight loss, ovulation rates, and androgen concentrations, bolstering the metabolic and reproductive benefits of orlistat use [86]. Sibutramine medication has also been shown to lower the waist-hip ratio and serum TG levels, suggesting that it may benefit obese PCOS patients [85]. Surgical approaches such as bariatric surgery in PCOS patients are increasingly being explored. A recent study demonstrated that the surgical modality for weight loss in severely obese PCOS patients resulted in a tremendous 96% resolution rate of PCOS (95% CI, 88-100%). However, the chances of recurrence are still not known [87]. Another study found that morbidly obese females with PCOS who had underwent biliopancreatic diversion or laparoscopic gastric bypass lost an average of 41 kg in the first year after surgery, with significant improvements in hyperandrogenism and cardiometabolic abnormalities [88]. Bariatric surgery is, thus, an emerging treatment modality for obese PCOS, which demands substantial research and, if proven to be helpful, after careful consideration of any potential risks, can be successfully implemented as a treatment option. 

Limitations

This article focuses on cardio-metabolic aspects only in the light of PCOS, ignoring other independent risk factors like smoking, menopause, stress, family history, and the multi-factorial causation of PCOS with genetic and environmental influences have also not been explored.

## Conclusions

From the data reviewed in this article, it is evident that PCOS is no longer limited to being a reproductive disorder and has many cardio-metabolic implications. This article has highlighted a plethora of cardiovascular and metabolic disturbances such as atherosclerosis, HTN, endothelial dysfunction, T2DM, and IGT, along with an outline of each of their pathogenesis, exploring the intricate relationship PCOS has with our bodily functions. According to all the mentioned studies, PCOS patients were at substantial risk of developing cardio-metabolic disorders in their lifetime compared to females unaffected by PCOS, and the disturbances, if identified early, can be prevented or treated to avoid further complications. This indicates the need for numerous studies and widespread screening procedures to help tackle this modern-era disease. However, a few studies have demonstrated no particular relation between cardio-metabolic outcomes and PCOS. In light of this disparity, further longer-term population-based studies among early reproductive age females have to be done to fill the knowledge gap on the long-term outcomes of cardiovascular and metabolic risk factors, and the females included in the studies ought to be followed up until their late menopause when most cardio-metabolic disturbances occur, which will provide us with the required information to understand the intricate systemic effects of PCOS. Furthermore, this article has also put forth various treatment modalities like diet, exercise, and drugs such as metformin and orlistat and their importance to help physicians tackle this enigmatic disease.
